# Laron Syndrome Research Paves the Way for New Insights in Oncological Investigation

**DOI:** 10.3390/cells9112446

**Published:** 2020-11-09

**Authors:** Haim Werner, Rive Sarfstein, Karthik Nagaraj, Zvi Laron

**Affiliations:** 1 Department of Human Molecular Genetics and Biochemistry, Sackler School of Medicine, Tel Aviv University, Tel Aviv 69978, Israel; rives@tauex.tau.ac.il (R.S.); mailkartz@gmail.com (K.N.); 2 Shalom and Varda Yoran Institute for Human Genome Research, Tel Aviv University, Tel Aviv 69978, Israel; 3 Endocrine and Diabetes Research Unit, Schneider Children’s Medical Center, Petah Tikva 49292, Israel; laronz@clalit.org.il

**Keywords:** growth hormone, growth hormone receptor, insulin-like growth factor-1 (IGF1), Laron syndrome, cancer protection

## Abstract

Laron syndrome (LS) is a rare genetic endocrinopathy that results from mutation of the growth hormone receptor (*GH-R*) gene and is typically associated with dwarfism and obesity. LS is the best characterized entity under the spectrum of the congenital insulin-like growth factor-1 (IGF1) deficiencies. Epidemiological analyses have shown that LS patients do not develop cancer, whereas heterozygous family members have a cancer prevalence similar to the general population. To identify genes and signaling pathways differentially represented in LS that may help delineate a biochemical and molecular basis for cancer protection, we have recently conducted a genome-wide profiling of LS patients. Studies were based on our collection of Epstein–Barr virus (EBV)-immortalized lymphoblastoid cell lines derived from LS patients, relatives and healthy controls. Bioinformatic analyses identified differences in gene expression in several pathways, including apoptosis, metabolic control, cytokine biology, Jak-STAT and PI3K-AKT signaling, etc. Genes involved in the control of cell cycle, motility, growth and oncogenic transformation are, in general, down-regulated in LS. These genetic events seem to have a major impact on the biological properties of LS cells, including proliferation, apoptosis, response to oxidative stress, etc. Furthermore, genomic analyses allowed us to identify novel IGF1 downstream target genes that have not been previously linked to the IGF1 signaling pathway. In summary, by ‘*mining*’ genomic data from LS patients, we were able to generate clinically-relevant information in oncology and, potentially, related disciplines.

## 1. The Growth Hormone-Insulin-Like Growth Factor 1 Endocrine Axis

The biological actions of the growth hormone (GH)-insulin-like growth factor-1 (IGF1) axis have been clearly delineated [1,2]. The role of IGF1 as the mediator of the GH-stimulated incorporation of sulfate into cartilage was demonstrated more than sixty years ago [3]. The specific, GH-activated serum factor, originally termed *sulfation factor* and then *somatomedin*, is now accepted as IGF1 [4]. The IGF system comprises two ligands, IGF1 and IGF2. The IGFs developed early in evolution, possibly as regulators of cellular proliferation in relation to nutrient availability [5,6]. At the cellular level, IGF1 functions as a progression factor required to traverse the various phases of the cell cycle [7,8,9,10]. Circulating IGF1 levels are dependent on liver production, which is tightly controlled by hypophyseal GH. In addition to its classic endocrine mode of action, a number of extrahepatic tissues display the biosynthetic machinery necessary to produce IGF1. These organs include the brain, kidney, stomach, and others [1,2,6]. The dependence of locally-produced IGF1 on GH stimulation is still a matter of debate.

IGF1 and IGF2 activate a common cell-surface receptor, the IGF1 receptor (IGF1R). Activation (i.e., phosphorylation) of the IGF1R tyrosine kinase domain by either IGF1 or IGF2 leads to the subsequent activation of a cytoplasmic enzymatic cascade that is directly responsible for the execution of the metabolic and growth actions of hormones [11,12]. The IGF1R is regarded as one of the most potent anti-apoptotic, pro-survival growth factor receptors [13,14,15,16]. The IGF2 receptor (IGF2R) is identical to the mannose 6-phosphate receptor, a cell-surface protein that binds IGF2 with high affinity and targets it for lysosomal degradation [17,18]. IGF2R lacks a tyrosine kinase domain and, therefore, is not involved in cell signaling. The activities of IGF1/IGF2 are modulated by a family of at least six IGF-binding proteins (IGFBPs) that carry the IGFs in the circulation and extracellular space [19,20].

The present review is aimed at dissecting the role of the GH–IGF1 axis in cancer biology. Our comprehensive analyses of Laron syndrome (LS) patients, a rare endocrinopathy associated with major growth deficits, generated novel insights that, we believe, might have translational relevance in oncology. As described below, gain-of-knowledge was made possible due to a number of recent technological and scientific breakthroughs, including the availability of genomic and proteomic platforms designed for the global exploration of massive amounts of data. The analyses presented here are ultimately aimed at delineating a molecular signature associated with cancer protection.

## 2. The GH-IGF1 Axis and Growth Retardation

Growth retardation in infants is multifactorial, although a large portion of cases remain *idiopathic* because no specific (genetic or other) defect can be identified [21]. Prenatal *IGF1* gene expression is GH-independent and becomes GH-dependent shortly before birth [2]. *IGF1* expression remains dependent on GH secretion during postnatal life. *Congenital IGF1 deficiencies* are characterized by low serum IGF1 but normal to elevated GH production [22]. These conditions result from: (i) a GH releasing hormone-receptor (*GHRH-R*) defect; (ii) *GH* gene deletion (isolated GH deficiency, IGHD); (iii) GH receptor (*GHR*) gene deletion or mutation (Laron syndrome); (iv) *IGF1* gene deletion or *IGF1R* gene defect. Additional conditions leading to congenital IGF1 deficiency are defects of post-GHR signaling (e.g., STAT5 defects) as well as a number of disorders associated with reduced IGF1 stability or availability, including acid-labile subunit (*ALS*) mutation and molecular defect of the metalloproteinase pregnancy-associated plasma protein A2 (PAPP-A2) [23,24,25,26,27,28,29,30].

The essential role played by the IGF system in growth and development is illustrated by the severe growth deficits observed in mice, in which various components of the IGF system, including ligands and receptors, were disrupted by homologous recombination [31,32]. Heterozygous mice for a disrupted *igf1* allele had a body weight at birth that was ~10–20% lower than that of wild-type animals. Homozygous *igf1* null mice weighed ~40% less than wild-type animals and exhibited a very high perinatal mortality rate as well as a number of phenotypic alterations. Genetic inactivation of the *igf1r* gene yields the most severe phenotype in this pathway, including neural, muscular, bone, and skin defects. Homozygous *igf1r* null mice die at birth as a result of respiratory failure caused by acute muscular hypoplasia [33].

To distinguish between the endocrine and autocrine/paracrine effects of IGF1 on growth and development, Yakar et al. generated mice with liver-specific deletion of the *igf1* gene [34]. Despite a major (~80%) reduction in circulating IGF1 levels, the overall growth of these animals was not different from that of their control littermates. These findings demonstrate that despite its minor contribution to the total IGF1 concentration in blood, locally-produced (extrahepatic) IGF1 has a critical role in growth and development. On the other hand, List et al. reported that liver-specific disruption of the *GHR* gene led to a major reduction in serum IGF1 levels and a stunted growth phenotype, especially later in life [35]. This phenotype was associated with increased local IGF1 production, altered body composition, and abnormal adipokine profiles. The differences between both animal models reflect the complexity of this hormonal system and the distinct impact of specific gene defects.

## 3. IGF1: A Validated Cancer Risk Factor

A high cellular turnover in epithelial tissues is generally regarded as a contributing factor to the propagation of a neoplasm [4]. The proliferative and antiapoptotic activities of IGF1 led to several observational studies that were aimed at elucidating the potential connection between circulating IGF1 levels and increased risk of cancer. Large epidemiological studies have suggested that high endocrine IGF1 levels are associated with an augmented risk for breast, prostate, lung, sarcoma, and colorectal cancer [36]. Specifically, in a prospective nested control study (the Nurse’s Health Study), the relative risk (RR) of breast cancer in premenopausal women was 4.6 in the upper tertile of IGF1 values in comparison to women in the lower tertile [37]. The RR increased to 7.3 when the concentrations of IGFBP3 (a protective factor) were included in the analysis. Likewise, results of the Physicians’ Health Study revealed that the RR to develop prostate cancer was 4.3 among men in the upper quartile of IGF1 values compared to individuals in the lower quartile [38]. It is noteworthy that IGF1 levels were measured an average of seven years before the diagnosis of prostate cancer. In addition to hormone-dependent prostate and breast carcinomas, the role of IGF1 as a risk factor was evaluated in various non-hormone-dependent malignancies. Analyses of colon cancer risk in the Nurse’s Health Study and the Physician’s Health Study showed an increased risk in individuals with the highest IGF1 values [39].

Several mechanisms have been suggested to explain the role of the IGF system in the initiation and/or progression of neoplasia [40]. Although IGF1 was shown to increase chromosomal fragility under experimental conditions [41], it is usually considered to be non-genotoxic. Whether the use of high doses of IGF1 induce DNA damage or mutations in humans has not been investigated. However, the proliferative actions of IGF1 have been well characterized. A biologically-based, computerized description of carcinogenesis suggested that an increase in cell proliferation could account for the carcinogenicity of non-genotoxic compounds [42]. Once a malignant transformation event has occurred, cell survival of already transformed cells depends on IGF1 action. The disruption of internal checks associated with the neoplastic phenotype is further emphasized by the finding that IGF1 action can override the cellular signals of apoptosis [7].

## 4. Laron Syndrome: A Prototypical Case of Congenital IGF1 Deficiency

Laron syndrome, or primary GH insensitivity (OMIM#262500), is an autosomal recessive hereditary disease caused by molecular defect of the *GHR* gene, leading to GH resistance and dwarfism. LS constitutes the best-characterized entity under the umbrella of congenital IGF1 deficiencies. Historical aspects related to the discovery of the disease as well as the impact of this syndrome on our current understanding of GH-IGF1 pathophysiology have been reported elsewhere [43,44,45,46]. The disease was originally identified in the late 1950s in Jewish patients of Yemenite origin [45]. Following the first report in 1966, LS patients of various ethnic origins were identified in various regions of the world [47]. Of interest, most of the patients were of Mediterranean, Middle Eastern, or South Asian origin, including a large cohort in Ecuador [48]. According to most estimates, the approximate number of diagnosed LS patients worldwide ranges between 400 and 500 individuals.

**Figure 1:**
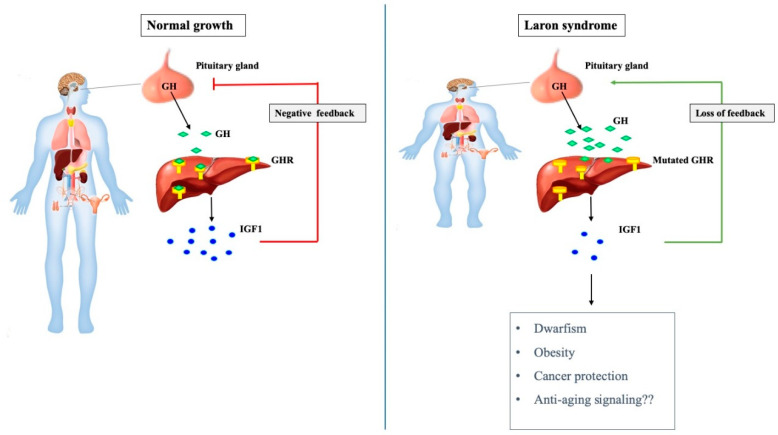
The growth hormone-IGF1 axis in normal and pathological growth. Pituitary-produced growth hormone (GH) stimulates insulin-like growth factor-1 (IGF1) secretion from the liver, leading to bone elongation and longitudinal growth (**left panel**). Laron syndrome (LS) is associated with mutation of the *GHR* gene, as a result of which the liver (and, most probably, additional extrahepatic tissue) is no longer able to produce IGF1 at physiological levels in response to GH stimulation (**right panel**). Abrogation of IGF1 biosynthesis leads to impaired growth and concomitant relaxation of negative feed-back regulation of GH production at the pituitary gland. Loss of inhibitory control results in very high circulating GH levels. In addition to short stature, LS is associated with obesity and protection from cancer. Enhanced longevity has been demonstrated in animal models of LS (‘Laron’ mouse).

Whereas the clinical history, appearance, and laboratory findings of the first LS patients resembled those described earlier in children with IGHD, a recently developed radioimmunoassay [49] revealed that their serum GH levels were very high, in the so-called *acromegalic range* [47]. Initial immunological studies established that the structure as well as the immunogenic properties of the circulating GH molecule were normal, as proven by radio-receptor assays using human liver membranes [50]. However, liver biopsies from two LS patients showed no specific binding of ^125^I-hGH to liver microsomes [51]. These laboratory findings provided the first evidence that LS patients have a defective GHR that causes GH insensitivity and diminished liver IGF1 production. As a result of the reduced endocrine IGF1 levels, there is a relaxation of the inhibitory *feed-back* regulatory loop of GH biosynthesis at the hypophyseal level, with ensuing augmentation of circulating GH levels (Figure 1).

The identification of an exon deletion or mutation of the *GHR* gene as the molecular defect underlying the etiology of LS was reported in 1989 [52,53]. Sixteen different molecular defects were found in the Israeli cohort, whereas the total number known today amounts to over 70 [54,55]. The majority of LS patients from the Ecuadorian cohort are homozygous for an A to G splice site mutation at position 180 in exon 6 of the *GHR* gene [55]. As the same mutation was found in a Jewish girl of Moroccan origin, it is assumed that the gene was brought to South and Central America by Sephardic Jews fleeing the Spanish Inquisition in the 15–16th centuries [56]. Despite variability in the mutational spectrum, the phenotypic consequences are remarkably similar, i.e., dwarfism and lack of GH signaling. The typical features of classical LS are short stature (−4 to −10 SDS below median height), typical face, obesity, high serum GH, and low to undetectable serum IGF1, unresponsive to the administration of exogenous GH [45]. An animal model of LS (the ‘*Laron*’ mouse) was engineered by disrupting the GH-binding protein gene [57]. Similar to humans, *Laron* mice have low IGF1 and high inactive GH levels.

The only effective treatment for LS patients is biosynthetic IGF1, available since 1986, which leads to acceleration of linear growth [58,59]. According to most clinical reports, the growth velocity during the first year of treatment is higher than in subsequent years. However, despite the effective stimulation of linear growth, the growth velocity is not as intense as that achieved with hGH in GH deficient patients [60]. With continuous treatment, there is also progressive growth of the extremities and a fast *catch-up* growth of the head circumference.

## 5. Laron Syndrome and Cancer Protection: Epidemiological Data

As alluded to above, individuals with high circulating IGF1 levels, as well as those with insulin resistance and/or obesity, are at increased risk for several types of cancer. Nevertheless, it is not clear whether IGF1 plays, by endocrine, paracrine, or autocrine mechanisms, a role in the *etiology* or only in the *progression* of neoplasms [61]. To provide a different perspective on the linkage between GH-IGF1 and cancer, prevalence of malignancies was recently assessed in a group of patients with congenital IGF1 deficiency [62,63]. The cohort investigated included 538 patients, divided into the following diagnostic groups: (i) LS (*n* = 230); (ii) IGHD (*n* = 116); (iii) *GHRH-R* mutations (*n* = 79); (iv) congenital multiple pituitary hormone deficiency (cMPHD) (*n* = 113). In addition, the analyses included 752 first-degree family members (out of which 274 were siblings) and 131 further relatives.

The analyses revealed that none of the 230 LS patients (up to the age of 85) had developed a malignancy, despite the fact that 66 of them had been treated with IGF1 and two had received hGH as well. Eighteen (8.3%) instances of malignancy were reported among 218 first-degree relatives, twenty-five (22.1%) cases were reported in 113 further relatives, and five (5.8%) tumors were reported in 86 siblings of LS patients. The differences between the prevalence of malignancies in LS versus first-degree relatives, further relatives, or siblings were regarded as statistically significant (Table 1).

Differences in cancer incidence between patients and relatives were also noticed in the other diagnostic groups. Specifically, out of the 116 patients with IGHD, only one boy (0.9%) who suffered from xeroderma pigmentosum was reported to have a basal cell carcinoma. Fifty-nine of the IGHD patients had been treated with hGH, including the boy who developed the carcinoma. In comparison, 3.4%, 30.8%, and 2.1% cases of cancer were reported in first-degree relatives, further relatives, and siblings, respectively, of IGHD patients. Out of the 79 patients with *GHRH-R* mutations (out of which twelve were previously treated with hGH), three (3.8%) had cancer (one of them treated with hGH) and so had three (2.7%) out of 113 patients with cMPHD. Epidemiological data are consistent with the concept that congenital IGF1 deficiency, or deficiency in early childhood, confers protection against future development of cancer. As mentioned above, this inference reaches statistical significance in LS patients. Similar trends (although not always reaching significance) were seen in the other pathologic entities. Reports of protection from cancer were also reported by Guevara-Aguirre and colleagues in the Ecuadorian cohort of LS patients [64]. In this population, tumors were not a main cause of death among patients who died before 1988. Moreover, there was no proof of cancer among 99 LS patients since 1988. Cancer frequency among relatives was similar to the Ecuadorian population (~20%). Of interest is that a double heterozygous LS patient developed an ovarian carcinoma (personal communication)

An additional aspect that deserves consideration is the fact that tumor spread might reflect immune escape of circulating malignant cells. Of notice, congenital IGF1 deficiencies were reported not to be associated with immune deficiency [45]. Data described in the following sections are consistent with the notion that cancer protection in LS is most probably not related to improved immune surveillance but rather to a reduction in the primary events leading to cancer development. Finally, epidemiological data are supported by animal experiments using the GH-R/GH-BP knockout (‘Laron’) mouse [65]. Specifically, crossing of the C3(1)T antigen mouse with the GH-R/GH-BP knockout resulted in markedly reduced prostate cancer development [66].

## 6. Genome-Wide Profiling of Laron Syndrome Patients

The association between life-long diminished IGF1 levels in congenital deficiencies and cancer protection highlights the central role of IGF1 in the etiology of malignancies. Recognition of this linkage prompted us to conduct a genomic profiling of LS patients aimed at identifying IGF1-dependent genes and signaling pathways that are differentially expressed in LS and that may shed information on the molecular foundation for cancer evasion in this condition. Studies were based on the collection of Epstein–Barr virus (EBV)-immortalized lymphoblastoid cell lines derived from LS patients, relatives, and healthy controls [67]. The collection is available at the Laboratory for the Genetics of Israeli Populations (Tel Aviv University, Israel). 

EBV-immortalized lymphoblastoid cell lines constitute an important tool in biomedical research [68]. Cell lines were generated from lymphocytes isolated from blood samples. Despite viral immortalization, the cells are regarded as non-carcinogenic. Furthermore, cells retain the genomic characteristics of the donors and are, therefore, perfectly suited for this type of analysis. Of importance, given that the cells are usually grown in a serum-containing medium, caution has to be exerted when extrapolating experimental data generated with these cells to the in vivo conditions. Moreover, comparisons with cancer-derived cells are not always feasible. In spite of these limitations, the use of EBV-immortalized lymphoblastoids had an enormous impact on genomic research.

RNA obtained from four female LS patients and four controls of the same age range (LS, 44.25 ± 6.08 yr; controls, 51.75 ± 11.3 y; mean ± SD; *p*-value = 0.29) and ethnic origin (Yemen, Irak, Iran) was used for genomic analyses. Affymetrix GeneChip^®^ Human Gene 1.0 ST Arrays, which offer whole-transcript coverage, were used. These arrays interrogate 28,869 well-annotated genes and non-coding RNAs and have greater than 99% coverage of sequences present in the RefSeq database.

**Figure 2:**
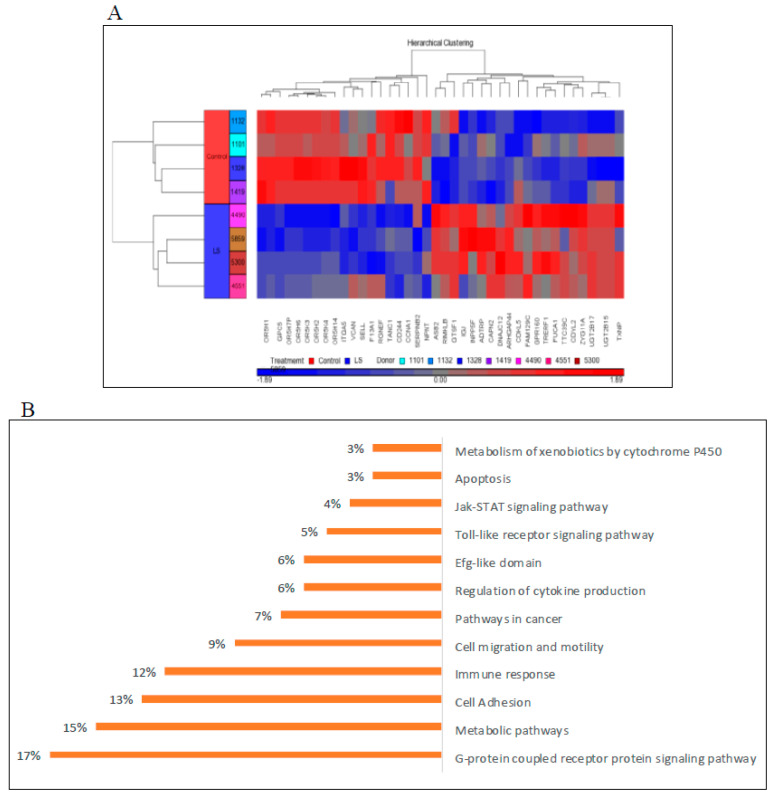
Genomic analysis of Laron Syndrome patients. (**A**) Hierarchical cluster analysis of differentially expressed genes in lymphoblastoids derived from four female LS patients and four controls of the same age range (LS, 44.25 ± 6.08 yr; controls, 51.75 ± 11.3 y) and ethnic (Yemen, Irak, Iran) origin. The figure depicts a cluster of 39 differentially expressed genes (FC > 2 or < than −2 and *p*-value < 0.05). Up-regulated genes are shown in red and down-regulated genes are shown in blue. FC, fold change. This panel was obtained from [67]. (**B**) Functional analysis of differentially represented signaling pathways in LS. Bioinformatic analyses were conducted using the *David* and *WebGestalt* platforms. The % values in each category correspond to the percentage of the total number of differentially expressed genes.

Hierarchical cluster analysis using the Partek Genomics Suite software led to the identification of thirty-nine annotated genes that were differentially expressed in LS (either up- or down-regulated) compared to controls (with a *p*-value of <0.05 and fold-change difference cutoff >|2|) (Figure 2A). Principal component analysis (PCA) revealed a good discrimination between experimental groups. The bioinformatic analyses described below highlight the power of post-genomic platforms to study previously unrecognized aspects of IGF1 biology.

## 7. Bioinformatic Analyses Identify New IGF1 Target Genes and Signaling Pathways

Comprehensive functional enrichment analyses were performed using the David (david.ncifcrf.gov) and WebGestalt (www.webgestalt.org) platforms. The aim of these studies was to find co-expressed genes sharing the same pathways. Our analyses identified a number of genes that are differentially expressed in lymphoblastoid cells of LS patients compared to healthy controls of the same gender, age, and ethnic group. This differential expression may, potentially, explain the evasion of LS patients from cancer. Bioinformatic analyses revealed that differences in gene expression occur in several pathways, including apoptosis, metabolic control, cytokine-cytokine receptor interaction, Jak-STAT and PI3K-AKT signaling, etc. A schematic diagram of differentially represented pathways in LS is presented in Figure 2B.

Analyses revealed that genes involved in the control of cell cycle, motility, growth, and oncogenic transformation were, for the most part, down-regulated in LS-derived, compared to control, cell lines. These genes include, among others, cyclin A1, cyclin D1, serpin B2, versican, and transcription factor Sp1 (Table 2). Sp1 has been identified as a key regulator of *IGF1R* gene transcription [15,16] and it is typically overexpressed in malignantly-transformed cells. Hence, bioinformatic data are consistent with the notion that life-long lack of exposure to endocrine (and, probably, locally-produced) IGF1 in LS might lead to down-regulation of genes, with a positive impact on survival and proliferation. This scenario is consistent with the view that IGF1 exposure activates epigenetic and transcription pathways that are critical for mitogenic gene expression. Diminished IGF1 levels in LS patients lead to relaxation of expression and/or activation of pro-survival, anti-apoptotic signaling pathways, with important consequences in terms of cancer avoidance.

Conversely, genomic analyses revealed enhanced expression of genes associated with protection from toxic xenobiotic substances and metabolites in LS-derived lymphoblastoid cells. Specifically, the UDP-glycosyltransferase (*UGT*) genes [69,70], which code for enzymes that convert xenobiotic and endobiotic substances into lipophilic compounds, thereby facilitating clearance from the body as part of a liver detoxification system, were several-fold higher in LS, compared to control, cells. Of special interest is the elevated expression of both *UGT2B15* and *UGT2B17* (11- and 7-fold, respectively) in LS. The UGT enzymes encoded by both genes facilitate the catabolism of steroid hormones involved in the etiology of certain cancers, in particular breast and prostate tumors. Thus, data point towards an enhanced capacity of LS cells to respond to a variety of exogenous cellular insults (e.g., xenobiotic substances) as well as endogenous metabolites (e.g., steroid hormones). The ability of LS cells to act in response to oxidative stress is described below.

Finally, genomic analysis identified *ZYG11A*, a member of the *ZYG11* gene family of cell cycle regulators, as a highly expressed (fold-change = 3.0 versus controls) gene in LS cells. Consistently, ZYG11A was inhibited by IGF1 in endometrial cancer cells in a p53-dependent manner [71]. In addition, bioinformatic analyses identified *nephronectin*, an intracellular and secreted extracellular matrix protein with important roles in kidney development, as the top down-regulated gene in LS cells (fold-change = −3.12 versus controls) [72]. Neither ZYG11A nor nephronectin have been previously linked to the IGF1-signaling pathway. The biological and clinical implications of these novel regulatory links merit further investigation. In particular, it will be of interest to elucidate the mechanistic events responsible for the linkage between *ZYG11A* and/or *nephronectin* dysregulated expression and cancer protection.

## 8. LS Cells Display Altered Mitogenic Properties

Following identification of genes and signaling pathways that are differentially represented in LS, it was of importance to establish the impact of these paths on the biological properties of LS cells. Above all, it was of critical relevance to address the mechanisms that might underlie cancer protection in this condition. The following questions were formulated:

(i) What are the mitogenic properties of LS-derived cells?
(ii) Are the different phases of the cell cycle altered in LS?
(iii) What is the apoptotic index of LS cells?
(iv) Do LS cells respond differently to oxidative stress?
(v) Is autophagic machinery involved in cancer evasion in LS?

**Figure 3:**
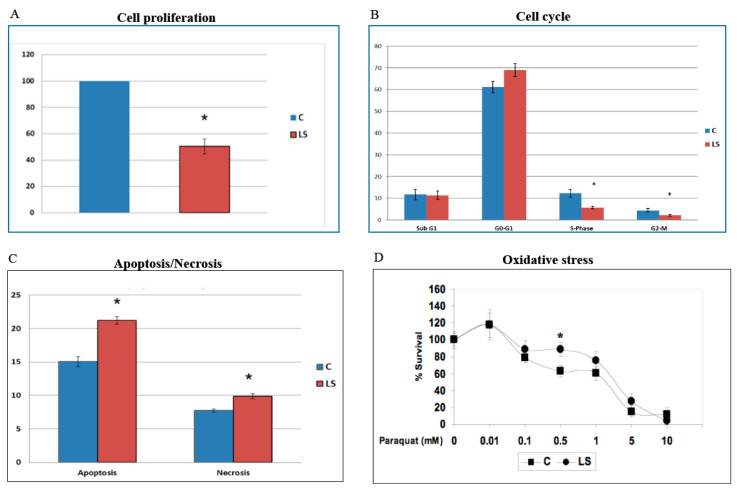
Analysis of biological functions in LS cells. (**A**) Cell proliferation. LS- and control-derived lymphoblastoid cells were maintained in a serum-free, IGF1-free medium for three days, after which proliferation was assessed using an XTT colorimetric kit. The statistical significance of differences between groups was assessed by a Student’s *t*-test. *, significantly different versus control (*p <* 0.05). (**B**) Cell cycle. Lymphoblastoids were exposed to etoposide (a DNA-damaging agent) for 24 h, after which cells were stained with propidium iodide and analyzed with a FACScalibur system to determine the percentage of cells in G0-G1, S, and G2-M phases. The graph presents the percentage of cells within each cell cycle phase. (**C**) Apoptosis/Necrosis. Basal apoptosis and necrosis were measured by flow cytometry analysis after staining cells with an Annexin-V antibody and propidium iodide. Necrotic cells were stained with propidium iodide and Annexin V, whereas apoptotic cells were stained only with Annexin V. (**D**) Oxidative stress. Cells were grown to confluence, after which the medium was changed to a fresh full medium in the presence of increasing doses of paraquat dichloride. Paraquat generates superoxide anion, which leads to the formation of toxic reactive oxygen species, such as hydrogen peroxide and hydroxyl radical, and the oxidation of cellular NADPH. Proliferation in response to oxidative damage was measured using an XTT kit. The graph depicts a pair of LS-derived and control cells, normalized for age and ethnic origin. A value of 100% was given to the cell number at time zero. Data described here were originally reported in [67].

XTT colorimetric assays revealed that the proliferation rate of LS-derived lymphoblastoid cells was reduced by 50% (Figure 3A) [67]. Cell cycle analyses showed that the proportion of cells in the G2-M phase in response to etoposide (a DNA-damaging agent) in LS was reduced by 50% in comparison to controls (2.2% in LS compared to 4.4% in control cells). Similarly, the proportion of LS cells in S phase was reduced in comparison to healthy cells. Combined, the data indicate that the response of LS cells to DNA damage in terms of cell cycle progression was attenuated (Figure 3B). Along the same lines, flow cytometry indicate that the percentage of apoptotic cells under basal conditions was 40% higher in LS compared to controls (Figure 3C). Likewise, the percentage of necrotic cells was increased by 27% in LS.

To examine the hypothesis that cancer protection in LS might be associated with enhanced resistance to oxidative damage, lymphoblastoids were treated with increasing doses of the oxidative agent *paraquat* for different time periods, after which cell survival was assessed. Results of XTT assays indicate that LS-derived lymphoblastoids display enhanced survivability in comparison to control cells over a broad range of paraquat concentrations (0.01–10 mM) (Figure 3D).

Autophagy is a major housekeeping mechanism, critically involved in the maintenance of normal cellular homeostasis [73]. This mechanism enables the clearance of damaged proteins and organelles via formation of special vesicles termed autophagosomes. The role of autophagy, however, extends beyond the removal of impaired cell components to many physiological and pathological processes, including oxidative stress and tumorigenesis. Proteins LC3β and P62 are involved in different aspects of autophagosome biology and are regarded as valid autophagy markers [74,75]. Western blots revealed that basal LC3β levels were reduced, whereas P62 values were elevated, in LS cells [67]. In addition, we discovered a major paraquat-induced increase in P62 levels in patient’s cells, suggesting the existence of a differentially-regulated autophagy machinery in LS. We assume that these autophagic adaptations correlate with enhanced survival of LS cells in response to oxidative stress.

## 9. Identification of Thioredoxin-Interacting Protein as a New Target for IGF1 Action

**Figure 4:**
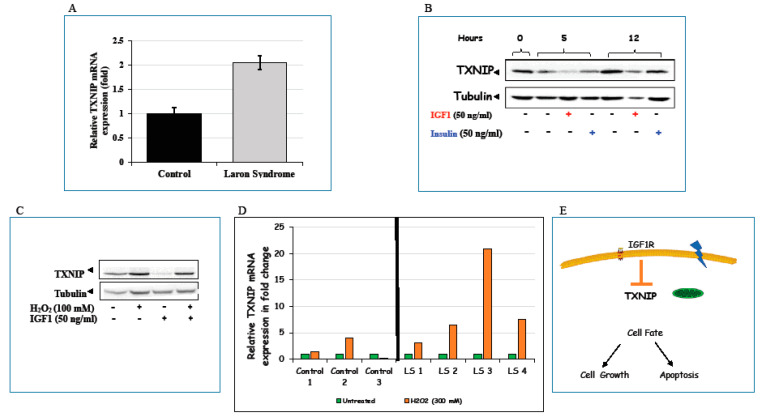
Regulation of thioredoxin-interacting protein (TXNIP) expression and action by IGF1. (**A**) Expression of TXNIP in LS cells. Total RNA was extracted from LS-derived and control lymphoblastoid cells and TXNIP mRNA levels were measured by RT-QPCR. (**B**) Effect of IGF1/insulin on TXNIP levels. HEK293 cells were starved for 24 h, after which they were treated with IGF1 or insulin (50 ng/mL) for 5 or 12 h. TXNIP levels were then measured by Western blots. (**C**) Effect of oxidative stress on TXNIP levels. Serum-starved HEK293 cells were treated with H_2_O_2_ (100 mM) or IGF1 or both for 2 h, after which TXNIP levels were measured by Western blots. (**D**) Effect of oxidative stress on TXNIP levels in LS-derived and control lymphoblastoid cell lines. Four individual LS-derived and three control lymphoblastoid cell lines were treated with 300 mM of H_2_O_2_ or left unstimulated. Cells were harvested after 2 h and levels of TXNIP mRNA were measured by RT-QPCR. A value of 1 was given to TXNIP mRNA levels in untreated cells (green bars). (**E**) Schematic representation of the regulation of TXNIP expression by IGF1. TXNIP is a metabolic gene that functions as an oxidative and glucose sensor. TXNIP mRNA levels were more than two-fold higher in LS-derived lymphoblastoid cells than in healthy control cells. IGF1 was shown to exert its anti-apoptotic effect via downregulation of TXNIP expression. Part of the data shown here was originally reported in [81].

Among other genes shown to be differentially represented in LS, the finding that thioredoxin-interacting protein (TXNIP) mRNA levels were more than two-fold higher in LS than in healthy control-derived lymphoblastoid cell lines is of special interest and identifies new, previously unrecognized connections between IGF1 and the mitochondrial machinery (Figure 4A). TXNIP belongs to the α-arrestin family [76] and was initially discovered as a vitamin D3-induced gene in leukemia [77]. TXNIP binds to the catalytic active center of reduced thioredoxin (TRX) and inhibits its expression and activity, highlighting the critical participation of TXNIP in redox regulation [78,79,80].

Consistent with its enhanced expression in a depleted IGF1 milieu (such as the one typical of LS), addition of exogenous IGF1 (as well as insulin, albeit with lower potency) reduced TXNIP levels in different cultured cells (Figure 4B). The effect of IGF1 on *TXNIP* gene expression was mediated at the level of transcription, as revealed by *TXNIP* promoter transfection assays [81]. Both oxidative and glucose stresses led to marked increases in TXNIP levels, which were abrogated by IGF1 treatment (Figure 4C). Finally, oxidative insult resulted in enhanced TXNIP expression in LS, but not control, lymphoblastoid cells (Figure 4D).

Given the fact that IGF1 is a major regulator of cell survival and in view of the finding that IGF1 inhibits the oxidative stress-induced TXNIP upregulation, we envision a scenario in which IGF1 could inhibit apoptosis by down-regulating TXNIP at the transcriptional level (Figure 4E). The finding that TXNIP levels are increased in response to oxidation in patient-derived, but not control, lymphoblastoid cells is of major translational relevance. Independent of redox regulation, TXNIP also functions as a regulator of glucose metabolism [82]. Finally, the recent report that TXNIP levels are increased in diabetes is consistent with the enhanced susceptibility of LS patients to develop type 2 diabetes mellitus [83].

## 10. Deregulated Expression of IGFBPs in LS Contributes to Cancer Protection

**Figure 5:**
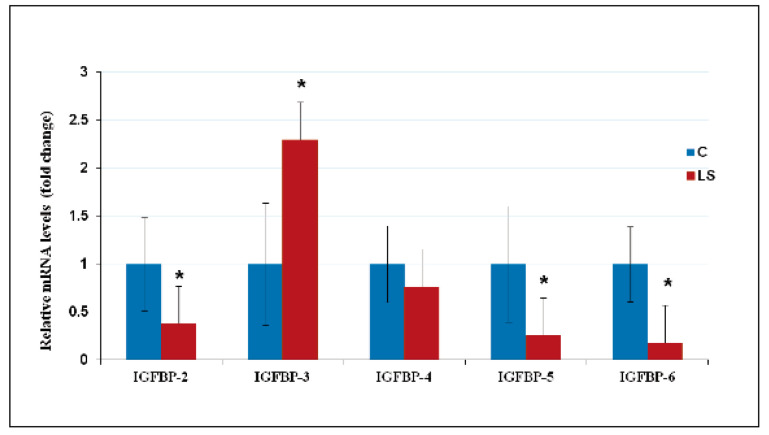
QRT-PCR analysis of IGF-binding protein (IGFBP) mRNA levels in LS cells. Lymphoblastoid cell lines derived from four LS patients (red bars) and four controls (blue bars) of the same age range, gender, and ethnic origin were harvested, after which total RNA was prepared and IGFBPs’ mRNA levels were measured by QRT-PCR. Primers employed are described in [84]. For each IGFBP mRNA, a value of 1 was given to the level exhibited by control cells. Bars denote mean ± SD (*n* = 4). *, *p* < 0.05 versus respective control. Data shown here were originally reported in [84].

Given the important role of IGFBPs in modulation of IGF1/IGF2 action [19], we investigated the impact of congenital IGF1 deficiency on IGFBPs expression [84]. QRT-PCR analyses revealed that IGFBP-2, -5, and -6 mRNA levels were reduced in LS-derived lymphoblastoids compared to those from healthy controls (Figure 5). Differences in mRNA levels were consistent with those at the protein level, as demonstrated by Western blots and confocal immunofluorescence. IGFBP-2 is most consistently described as pro-tumorigenic and it is reported to increase T-cell proliferation [85]. The decrease in IGFBP-2 levels in LS cells is, therefore, consistent with a protective action against cancer. IGFBP-5 has also been shown to promote T-cell migration [86] and IGFBP-6 has recently been shown to be a chemotactic agent [87]. The involvement of these specific IGFBPs in cancer protection and, in particular, their potential effects on tumor immunity merit further investigation.

On the other hand, basal IGFBP-3 levels were higher in LS than in control lymphoblastoid cultures. In view of the fact that IGFBP-3 generally functions as an anti-proliferative binding protein, increased IGFBP-3 levels may contribute to cancer protection in LS [88]. Studies have shown that adult and pediatric subjects with LS have decreased circulating IGFBP-3 levels [89,90]. This decrease is expected since IGF1 is a major positive regulator of IGFBP-3 stability. Thus, administration of IGF1 to LS patients led to increased plasma IGFBP-3 levels [91,92]. The discrepancy between in vitro and in vivo studies probably stems from the fact that lymphoblastoids were maintained in serum- (and IGF1-) containing medium. As mentioned in Section 6, EBV-immortalized lymphoblastoid cell lines do not always replicate the in vivo situation and caution must be exerted when analyzing the data generated with this experimental system.

Finally, experiments were conducted to assess IGFBPs’ responses to oxidative stress [84]. Data indicate that IGFBP-3 and -6 levels in LS were markedly reduced upon H_2_O_2_ treatment in comparison to control cells. This differential pattern was correlated with increased apoptosis in LS cells following oxidative damage. It is reasonable to assume that enhanced apoptosis may constitute a protection mechanism that prevents survival of damaged cells.

## 11. Interactions between Tumor Suppressor p53- and IGF1-Signaling Pathways

The biological interaction between the IGF1 and p53 pathways has been a topic of major interest in oncology [93]. Given the key role of p53 in the DNA damage response, analysis of the interplay between p53–IGF1 is expected to have major biological ramifications. p53 is activated in response to a wide spectrum of cellular stress signals, including DNA damage and telomere shortening, hypoxia, inflammation, and, finally, activation of oncogenes by mutations [94]. Accumulation of mutations constitutes an early event in malignant transformation and may lead to the establishment of a tumor [95]. p53-mediated cell cycle arrest enables damaged DNA to be repaired before the replicative phase of the cell cycle.

**Figure 6:**
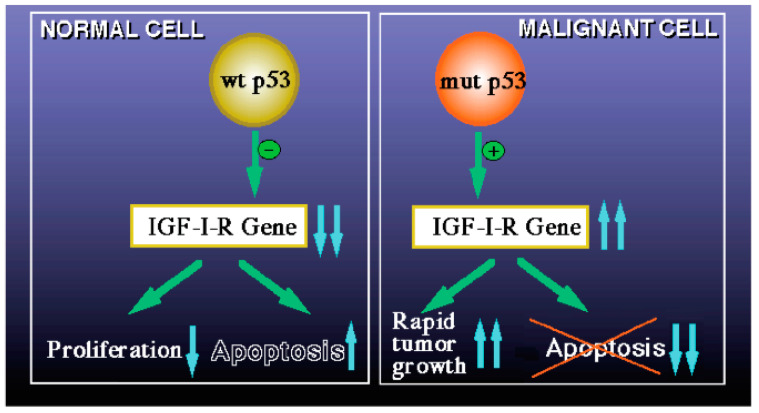
Schematic diagram of the regulation of *IGF1R* gene expression by wild-type and mutant p53. The mechanism of action of wild-type p53 involves transcriptional suppression of the *IGF1R* gene (**left panel**). Reduced IGF1R levels favor a post-mitotic, terminally-differentiated phenotype. Mutation of the *p53* gene in tumor cells disrupts its inhibitory activity, generating oncogenic molecules capable of transactivating the *IGF1R* gene (**right panel**). The abundance and activity of p53 itself is regulated by IGF1, which induces p53 degradation in an Mdm2-dependent fashion.

We have generated evidence that the mechanism of action of wild-type p53 involves transcriptional suppression of the *IGF1R* gene [96,97,98]. Mutation of p53 in tumor cells disrupts its inhibitory activity, generating oncogenic molecules capable of transactivating the *IGF1R* gene [16]. Lack of *IGF1R* inhibition by mutant p53 molecules may help expand cancer cell populations that are otherwise destined to die. The p53 homologues p63 and p73 exhibit some of the biological properties of p53, including the ability to recognize and bind p53 target sequences, transactivate p53-responsive genes, and induce apoptosis [99] (Figure 6).

Studies have suggested a convergence of the p53 and IGF1 signaling paths that results in a *feed-back* regulatory loop. IGF1 induces p53 degradation in an Mdm2- (an ubiquitin ligase) dependent manner, while Mdm2 physically associates with IGF1R and causes its ubiquitination and degradation [100,101,102]. Very low IGF1 concentrations in LS may result in high cellular p53 levels, a key element of the genome protection machinery.

## 12. Conclusions

The finding that homozygous LS patients do not develop cancer (at least up to the age of 85) is of exceptional clinical and scientific value. The epidemiological data presented here confirm the hypothesis that the GH–IGF1 axis has a fundamental role in shaping a cell’s decision on whether to adopt an apoptotic or, alternatively, oncogenic path. Our comprehensive analyses demonstrate that life-long lack of exposure to IGF1 in LS activates apoptotic, autophagic, and cancer-protecting pathways at the organism level. Concomitantly, reduced IGF1 signaling might have a major impact on nutrient sensing and response to oxidative stress.

By ‘*mining*’ genomic data from LS patients, a rare condition associated with cancer protection, we were able to generate clinically-relevant novel information and to translate this evidence into new avenues of research in oncology. We believe that this ‘*experiment of nature’* will prove useful in other areas of investigation, including metabolism, nutrition, aging, and longevity. Future mechanistic analyses of newly identified IGF1 target genes will help unravel the connection between the IGF1 pathway, cell metabolism, and cancer protection.

## Figures and Tables

**Table 1:** Epidemiological analyses of cancer prevalence in Laron syndrome patients. Prevalence of malignancies in LS and relatives was assessed by replying to a questionnaire that was filled-in by the primary care physician (Adapted from [63]).

	Laron Syndrome	First-Degree Relatives	Further Relatives	Sibilings Only
Total number (n)	230	218	113	86
Malignancies (number of events)	0	18	25	5
Prevalence of malignancy (%)	0	8.3	22.1	5.8
*p*-value (versus LS)		<0.001	<0.001	0.005

**Table 2:** Differentially-regulated genes in Laron syndrome. Bioinformatic analyses identified sets of over- and under-represed genes in LS-derived lymphoblastoid cells that might underlie cancer protection in this condition.

Genes up-Regulated in LS	Biological Role
UDP glucuronosyltransferase 2 family	Elimination of xenobiotic substances
G-protein coupled receptor	Signaling
Thioredoxin interacting protein	Metabolic regulation
ZYG11A	Cell cycle regulator
CAPN2	Extracellular matrix disassembly
**Genes down-regulated in LS**	**Biological role**
Cyclin A1	Cell cycle
AKT3	Apoptosis
Versican	Extracellular matrix proteoglycan
Olfactory receptor	Detection of odor molecules
Nephronectin	Cell adhesion
Serpin B2	Apoptosis and proliferation
Sp1	Transcription factor
